# Robotic-Assisted Sentinel Lymph Node Mapping With Indocyanine Green in Pelvic Malignancies: A Systematic Review and Meta-Analysis

**DOI:** 10.3389/fonc.2019.00585

**Published:** 2019-07-02

**Authors:** Yuqing Wu, Jibo Jing, Jinfeng Wang, Bin Xu, Mulong Du, Ming Chen

**Affiliations:** ^1^Department of Urology, Affiliated Zhongda Hospital of Southeast University, Nanjing, China; ^2^Surgical Research Center, School of Medicine, Institute of Urology, Southeast University, Nanjing, China; ^3^Department of Urology, School of Medicine, Affiliated Yancheng Hospital, Southeast University, Yancheng, China; ^4^Jiangsu Key Laboratory of Cancer Biomarkers, Department of Environmental Genomics, Prevention and Treatment, Collaborative Innovation Center for Cancer Personalized Medicine, Nanjing Medical University, Nanjing, China; ^5^Department of Biostatistics, Center for Global Health, School of Public Health, Nanjing Medical University, Nanjing, China

**Keywords:** indocyanine green, robotic surgery, pelvic, sentinel lymph node, cancer

## Abstract

**Objective:** Newer technologies such as near-infrared (NIR) imaging of the fluorescent dye indocyanine green (ICG) and daVinci Xi Surgical System have become promising tools for sentinel lymph node (SLN) mapping. This meta-analysis was conducted to comprehensively evaluate the diagnostic value of SLN in assessing lymph nodal metastasis in pelvic malignancies, using ICG with NIR imaging in robotic-assisted surgery.

**Materials and Methods:** A literature search was conducted using PubMed for studies in English before April 2019. The detection rate, sensitivity of SLN detection of metastatic disease, and factors associated with successful mapping (sample size, study design, mean age, mean body mass index, type of cancer) were synthesized for meta-analysis.

**Results:** A total of 17 articles including 1,059 patients were finally included. The reported detection rates of SLN ranged from 76 to 100%, with a pooled average rate of 95% (95% CI: 93–97; 17 studies). The sensitivity of SLN detection of metastatic disease ranged from 50 to 100% and the pooled sensitivity was 86% (95% CI: 75–94; 8 studies). There were no complications related to ICG administration reported.

**Conclusions:** NIR imaging system using ICG in robotic-assisted surgery is a feasible and safe method for SLN mapping. Due to its promising performance, it is considered to be an alternative to a complete pelvic lymph node dissection.

## Introduction

Pelvic lymphadenectomy (PLND), which remains the most accurate procedure for the detection of lymph node metastasis (LNM) in malignant pelvic tumors, plays an important role in surgical management of endometrial and prostate cancers (1, 2). However, the data from a prospective study suggests that lymphadenectomy is associated with the increasing operative time, blood loss and risk for surgical morbidity (e.g., blood vessel and nerve damage, lymphedema, and lymphocyst formation) (3, 4). Thus, novel nodal assessment techniques should be developed to improve the accuracy of LNM detection with lower surgical morbidity.

The biopsy of SLN which is defined as the first node to receive the drainage from the primary tumor, has been described by Canbanas in 1977 (5). The utility of SLN mapping can avoid the unnecessary LND when the SLN turns out to be negative (6). The different methods used in SLN mapping, such as blue dye, technetium, and ICG with NIR imaging have been investigated, among which ICG has been used clinically for over two decades with an excellent safety profile (7). Also, as one of four fluorochromes approved by US Food and Drug Administration, ICG may be of significant use in pelvic surgery due to its properties (8).

The NIR fluorescence imaging system in daVinci Xi Surgical System (Intuitive Surgical, Sunnyvale, CA, USA) with Firefly technology provides intraoperative ICG near-infrared fluorescence, especially for ICG at low concentrations in lymphatic mapping. While the high concentrations of ICG can be seen directly in green in color on a background of a grayscale image. Moreover, it brings surgeons great convenience to control the scope completely which the infrared and visible light systems are built in.

Nevertheless, although ICG-NIF imaging in SLN detection appears to be superior, there are few studies of meta-analysis on SLN mapping outcomes, and most of them focused on specific one or two types of cancer, especially on endometrial and cervical cancer. Thus, we performed this meta-analysis to evaluate the detection rates and sensitivity of SLN mapping in malignant pelvic tumors, including endometrial, cervical, bladder and prostate cancers.

## Materials and Methods

### Search Strategy

The literature search was conducted on PubMed, only English language studies before April 2019 included. The search terms used are as follow: (robotic OR robot) AND (indocyanine green OR ICG) AND lymph. In addition, the references of included studies were reviewed as supplement.

### Inclusion and Exclusion Criteria

Studies were included with the following inclusion criteria: (1) At least 10 patients diagnosed with pelvic malignancies; (2) Robotic-assisted surgery as the surgical approach; (3) Pelvic with or without other lymph node dissection as reference standard; (4) Pathological examination was taken, including hematoxylin-eosin (H&E) staining, immunohistochemistry (IHC) or ultrastaging; (5) ICG was used for SLN mapping; (6) Reported detection rate of SLN. The studies published as reviews and case reports were excluded.

### Study Quality Assessment

The quality of enrolled studies was assessed using the QUADAS-2 (Quality Assessment of Diagnostic Accuracy Studies-2) (9) tool by two reviewers independently. The items are shown in the Appendix Table 1 (Supplementary Material).

### Data Extraction

The following items were collected from each article: (1) authors; (2) year of publication; (3) sample size; (4) study design; (5) type of cancer; (6) injection site; (7) reference standard; (8) pathology assessment; (9) mean patient age and body mass index (BMI); (10) available outcome data.

The overall detection rate was estimated as the proportion of patients with at least 1 SLN identified among all the patients going though PLND. When assessing sensitivity and specificity, the patients who failed in SLN mapping were excluded, and sensitivity is defined as the percentage of patients with positive SLN divided by all patients with positive lymph nodes. The specificity is defined as the percentage of patients with negative SLN divided by all patients with negative lymph nodes. Studies in which the calculation of sensitivity was based on the number of removed node packets but not on the patients were excluded during assessment.

### Statistical Analysis

The Stata 15.0 and meta-disc were used to conduct all data analysis. The overall detection rate was calculated using a random-effects model under meta-analysis. The sensitivity of detection rates of SLN was evaluated using summary receiver operating characteristic curve (SROC). The I^2^ index was used to detect the heterogeneity among the studies. The Funnel plots, Egger's regression intercepts were used for the evaluation of publication bias. The univariate meta-regression was applied for the association of SLN detection rate and study characteristics, including sample size, study design, mean age, mean BMI, and type of cancer.

## Results

### Characteristics of Enrolled Studies

**Figure 1:**
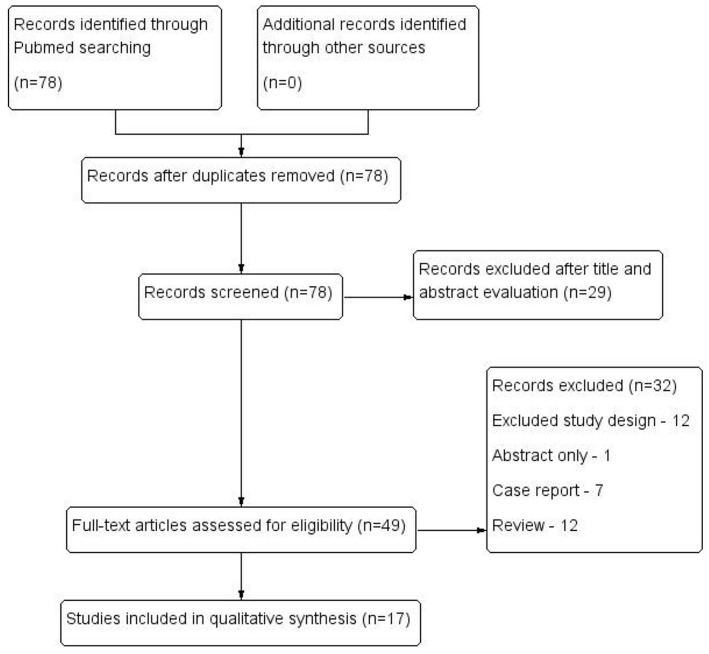
Flow diagram of studies identified, included, and excluded.

Of the 78 abstracts screened, 17 articles including 1,059 patients with pelvic malignancies were eligible for inclusion as demonstrated in Figure 1. The sample size of each study ranged from 10 to 197. SNL was evaluated for endometrial cancer in 9 articles, cervical cancer in 1, prostate cancer in 3 and bladder cancer in 1, with the other 3 articles dedicated in both endometrial cancer, and cervical cancer (10–26) (Table 1).

**Table 1:** Characteristics of included studies.

**Author, year**	**Sample size**	**Study design**	**Type of cancer**	**Injection site**	**Mean age**	**Mean BMI (kg/m^**2**^)**	**Mean number of SLNs detected**	**Mean number of non-SLNs removed**	**Reference standard**	**Pathology assessment**	**Detection rate**	**Sensitivity**
Rossi et al. (10)	20	Prospective	Endometrium/cervix	Cervical	61	31	4.5	23.5	Pelvic and para-aortic LND by guidelines	NR	85.0%	50.0%
Holloway et al. (25)	35	Retrospective	Endometrium	Cervical	63	33.1	NR	NR	Complete pelvic and common-iliac LND, aortic LND in high-grade EC	H&E, IHC, ultrastaging	100.0%	90.0%
Manny et al. (11)	50	Prospective	Prostate	Prostate	66	NR	NR	NR	Extended PLND	NR	76.0%	100.0%
Jewell et al. (12)	197	Retrospective	Endometrium/cervix	Cervical	60	30.2	3	NR	Pelvic and para-aortic LND by guidelines	H&E, ultrastaging	95.0%	NR
Manny et al. (26)	10	Prospective	Bladder	Bladder	71	NR	NR	NR	Complete pelvic and peri-aortic LND	NR	90.0%	100.0%
Sinno et al. (13)	38	Prospective	Endometrium	Cervical	65	31.1	4.8	NR	Complete pelvic and para-aortic LND if preoperative grade 3 endometrioid, serous, clear cell, or carcinosarcoma	H&E, ultrastaging	92.1%	100.0%
Paley et al. (14)	123	Prospective	Endometrium	Cervical	63	32	3	NR	Pelvic and peri-aortic LND if high risk	H&E	96.7%	100.0%
Ehrisman et al. (15)	20	Retrospective	Endometrium	Cervical	67	32.3	2	22	Complete pelvic LND or Memorial Sloan Kettering algorithm	H&E	90.0%	NR
Chennamsetty et al. (16)	20	Prospective	Prostate	Prostate	64	NR	5	NR	Extended PLND	NR	100.0%	NR
Beavis et al. (17)	31	Retrospective	Cervix	Cervical	43	26.5	4	14	Complete pelvic LND, para-aortic LND at surgeon discretion	H&E, IHC, ultrastaging	100.0%	100.0%
Hagen et al. (18)	108	Prospective	Endometrium	Cervical	66	27.5	NR	NR	Surgeon-discretion LND or Memorial Sloan Kettering algorithm	H&E, ultrastaging	96.0%	NR
Erikssion et al. (19)	56	Retrospective	Endometrium/cervix	Cervical	62	30.6	3	NR	Memorial Sloan Kettering algorithm	H&E, ultrastaging	95.0%	NR
Mendivil et al. (20)	87	Retrospective	Endometrium	Cervical	62	32.9	2	NR	Complete pelvic LND, para-aortic LND if at high risk	H&E	96.5%	NR
Harke et al. (21)	59	Prospective	Prostate	Prostate	64	NR	9	15	Extended PLND	NR	94.9%	78.0%
Rajanbabu and Agarwal (22)	69	Prospective	Endometrium	Cervical	60	27.9	5	NR	Pelvic and para-aortic LND by guidelines	H&E	95.7%	70.0%
Renz et al. (23)	90	Retrospective	Endometrium	Cervical	61	31	2	19	Complete pelvic LND, para-aortic LND by guidelines	H&E	88.0%	83.3%
Rozenholc et al. (24)	46	Prospective	Endometrium	Cervical	64	45	2.4	NR	Pelvic and para-aortic LND by guidelines	H&E, ultrastaging	89.1%	100.0%

*NR, not reported; H&E, hematoxylin-eosin; IHC, immunohistochemistry; BMI, body mass index; LND, lymph node dissection; PLND, pelvic lymph node dissection*.

An overall mean of 3.5 SLNs was removed per patient (13 studies). All the studies conducted PLND with or without para-aortic LND, and the mean non-SLNs identified per person was 17.9 (5 studies). The mean age of 1,059 patients was 62 years (17 studies) and mean BMI was 31.1 kg/m^2^ (13 studies) (Table 1). No complications, among all 17 articles, were described related to ICG administration.

### Data Analysis for the Detection Rate and Diagnostic Accuracy of SLN Mapping

**Figure 2:**
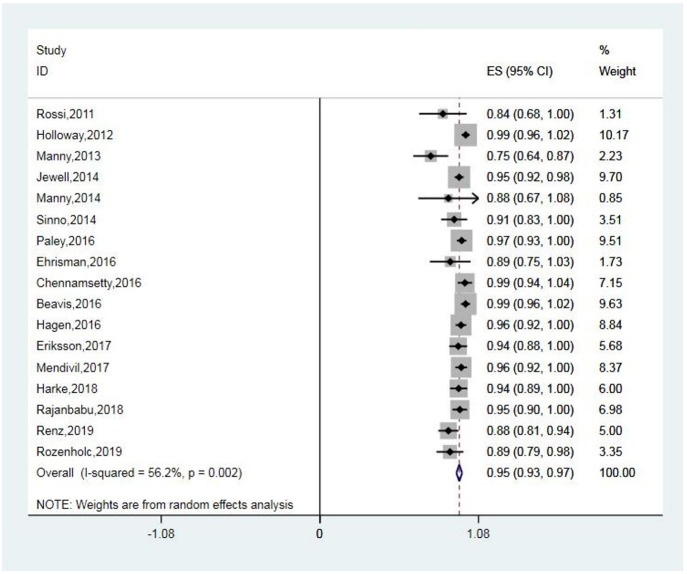
Forest plot of pooled overall detection rate and 95% CI in SLN mapping. CI, confidence interval; SLN, sentinel lymph node.

**Figure 3:**
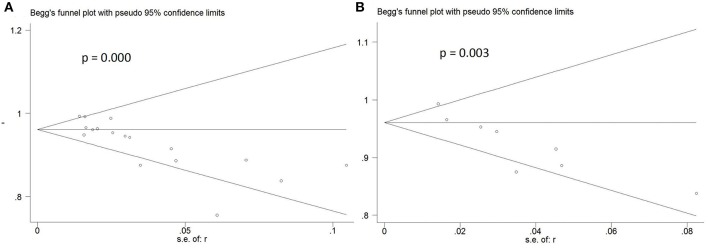
Funnel plot of pooled detection rate and sensitivity. **(A)** Funnel plot of pooled overall detection rate. **(B)** Funnel plot of pooled sensitivity.

The detection rates of SLN ranged from 76 to 100%, with a pooled average of 95% (95% CI: 93–97; 17 studies) with heterogeneity *I*^2^ = 56.2% (Figure 2). The funnel plot of the pooled overall SLN detection rate is shown in Figure 3. The Egger's regression intercept was −4.65 (*p* = 0.000).

**Figure 4:**
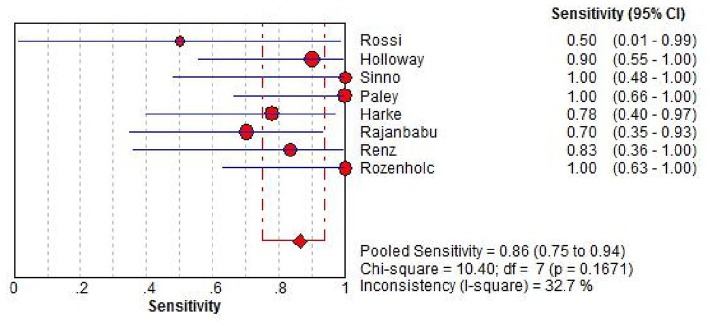
Forest plot of pooled sensitivity of SLN detection and 95% CI in SLN mapping. CI, confidence interval; SLN, sentinel lymph node.

**Figure 5:**
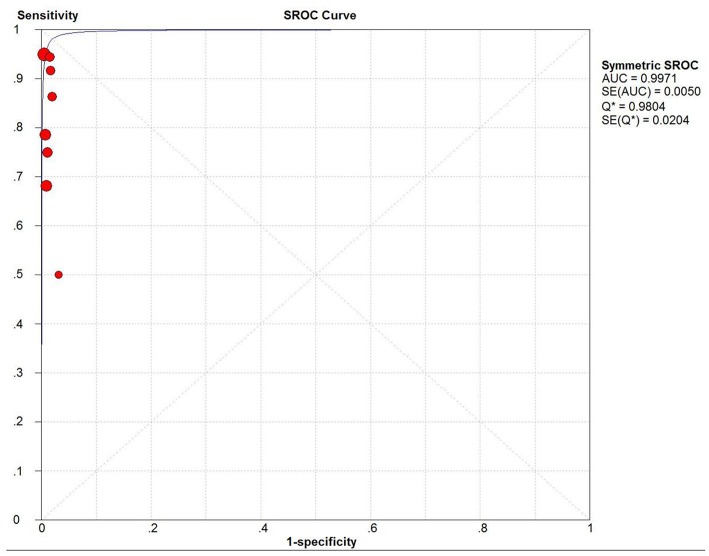
SROC curve. AUC, area under SROC curve; Q* indicates the point at which sensitivity = specificity.

Sensitivity of SLN mapping ranged from 50 to 100%. The pooled sensitivity of SLN detection of metastatic disease was 86% (95% CI: 75–94; 8 studies) (Figure 4). The funnel plot of the pooled sensitivity is shown in Figure 3. The Egger's regression intercept was found out to be −4.85 (*p* = 0.003). The pooled specificity and diagnostic odds ratio were 1.00 (95%Cl: 0.99–1.00) and 381.92 (95%Cl: 111.19–1311.85). The combined positive likelihood ratio and negative likelihood ratio were 67.31 (95% CI: 25.08–180.64) and 0.25 (95% CI: 0.15–0.40), respectively, [Appendix Figure 1 (Supplementary Material)]. According to SROC curve, AUC is found to be 0.9971 which is close to 1, showing the high value of ICG in diagnosing lymph node metastasis in pelvic malignancies (Figure 5).

### Test of Heterogeneity

Due to the heterogeneity *I*^2^ was found to be 56.2% in detection rate a sub-group analysis was conducted to find the reasons for the observed heterogeneity. Univariate meta-regression of SLN detection rate and study characteristics showed that study size, study method, mean patient age, mean patient BMI, and type of cancer were not significantly associated with detection rates (Table 2).

**Table 2:** Univariate meta-regression of SLN detection rate and study characteristics.

**Characteristics**	**Detection rate% (95% Cl)**	***P*-value**
**Sample size**		
<60	0.94 (0.91–0.97)	0.891
≥60	0.95 (0.93–0.97)	
**Study design**		
Prospective	0.94 (0.91–0.97)	0.456
Retrospective	0.96 (0.93–0.99)	
**Mean age**		
<63	0.95 (0.92–0.98)	0.985
≥63	0.95 (0.92–0.98)	
**Mean BMI**		
<32 kg/m^2^	0.95 (0.92–0.97)	0.475
≥32 kg/m^2^	0.96 (0.94–0.99)	
**Type of cancer**		
Uterus	0.95 (0.94–0.97)	0.561
Urology	0.91 (0.82–1.00)	

*CI, confidence interval; SLN, sentinel lymph node; BMI, body mass index*.

## Discussion

SLN mapping has been the standard of care for breast cancer and melanoma for a long time (27) and achieved success in many other types of cancer. During the process of assessing the value of SLN mapping, detection rate is taken into consideration in the very first place. Most of meta-analyses on diagnostic efficacy of SLN mapping were focused on uterine cancers, and to our knowledge, this is the first meta-analysis of that in pelvic malignancies.

It should be mentioned that in these previous meta-analyses, studies using both robotic-assisted system and indocyanine green fluorescence tracer in the meantime have not been analyzed statistically before. Compared with prior meta-analyses which studied on tracers including blue dye, ICG and/or 99mTc in endometrial cancer (28–30), this meta-analysis with only ICG used as tracer shows higher detection rate of SLN mapping with detection rate of 95% (95% CI: 93–97), and in study of Lin et al. the detection rate was only 76% (95%: 71–81) with blue dye alone. In their study, detection rates were of 93 and 86% in ICG and 99mTc combined with blue dye, respectively, (28). Moreover, in study of Smith et al. detection rates were found higher of 90.3% in ICG vs. 81% in blue dye (31).

Hybrid image-guided surgery technologies are increasingly gaining interest, such as combined radio- and fluorescence-guidance. In study of KleinJan et al. use of the hybrid tracer ICG-99mTc-nancolloid was evaluated and the detection rate was over 95% (32). Compared with the conventional radioguided SN approach, the additional cost of ICG-99mTc-nancolloid is negligible (33), and use of ICG involves only minor additional costs (34). According to prior studies, the use of ICG also brings several advantages, such as fewer adverse effects, less pain, and quicker transcutaneous real-time visualization (35, 36).

In this meta-analysis, the pooled sensitivity of SLN detection of lymph node metastasis was 86% (95% CI: 75–94). In study of SLN mapping by Rossi et al. there were only two patients with positive lymph nodes in those who had successful mapping and one of them showed negative SLN, leading to the lowest sensitivity of 50% (10). In other 10 studies with available data, 6 out of them showed the sensitivities of 100% and all of them were over 70% [(11–14, 17, 21–24, 26); Table 1]. In previous meta-analyses by Lin et al. and Smith et al. in uterine cancers with several tracers included, sensitivities were found 91% (95% Cl: 87–95) and 96% (95% CI: 93–98), respectively (28, 31).

In pilot meta-analysis, robotic-assisted surgery demonstrated higher detection rates than other modalities. The pilot study conducted by Lin et al. showed that robotic-assisted surgery led to 86% detection rate, when laparoscopy and laparotomy got that of 82 and 77%, respectively, in patients with endometrial cancer (28). In the literature before, a higher BMI is linked to lower detection rate of the SLN (13). However, in the study by Rozenholc et al. there was no difference in the detection rate between surgeries that were robotic (mean BMI 44.6) and laparoscopic (mean BMI 29.4) (24). Moreover, when compared with open surgery, robotic-assisted surgery results in fewer blood transfusions and leads to a slightly shorter hospital stay (37).

However, due to the limitations of robotic-assisted surgery, such as higher costs and the lack of haptic feedback, the current robotic technology has not become the standard technique of minimally invasive surgery worldwide. A single robotic surgical system can set a hospital back by about $2 million, and that's just to get started. Some of the instruments are disposable and need to be continually replaced. Additionally, the systems require regular maintenance at rates that can exceed $100,000, which limits the number of hospitals that can buy Da Vinci system (https://www.drugwatch.com/davinci-surgery/).

In a survey of complications of ICG angiography in Japan, the results indicated that indocyanine green was as safe as fluorescein (38), which was reported only 0.05% frequency of severe adverse reactions, such as circulatory shock, bronchospasm, laryngospasm, cardiac arrest, myocardial infarction, and tonic seizure (39).

Our systematic review and meta-analysis has limitations as follow. First, we only included English studies, which may lead to a potential language bias. Second, we didn't have individual patient data, such as age, BMI and so on. The results presented in this study were based on unadjusted estimates. Third, the number of studies included is not sufficient enough so that some subgroup analyses cannot be conducted, and it may contribute to a publication bias.

In conclusion, the NIR imaging system in robotic-assisted surgery with ICG dye is quite easy to master, and the present results confirmed that SLN mapping using ICG alone is a reliable and safe approach that performs well diagnostically when assessing lymph nodal metastasis in pelvic malignancies. Although it is considered to be an alternative to a complete pelvic lymph node dissection, studies with larger patient samples are still needed, especially in urology cancers like prostate and bladder cancer.

## Data Availability

All datasets generated for this study are included in the manuscript/Supplementary Files.

## Author Contributions

YW and BX: contributed conception and design of the study. JJ: organized the database. YW and JW: performed the statistical analysis. YW and JJ: wrote the first draft of the manuscript. JW, BX, MD, and MC: wrote sections of the manuscript. All authors contributed to manuscript revision, read, and approved the submitted version.

### Conflict of Interest Statement

The authors declare that the research was conducted in the absence of any commercial or financial relationships that could be construed as a potential conflict of interest.

## Abbreviations

SLN: sentinel lymph node
ICG: indocyanine green
NIR: near-infrared
LND: lymph node dissection
PLND: pelvic lymphadenectomy
CI: confidence interval.
