# Supplementary material for: Robotic-Assisted Sentinel Lymph Node Mapping With Indocyanine Green in Pelvic Malignancies: A Systematic Review and Meta-Analysis
Source: Front Oncol. 2019 Jul 2;9:585. doi: 10.3389/fonc.2019.00585 (PMC6614336; doi:10.3389/fonc.2019.00585)
Supplement: Supplementary file 1 [file Table_1.DOCX]

**Appendix Table 1.** Quality assessment of included studies

|  | Patient selection | | | Reference standard | | | Flow and timing | | |
| --- | --- | --- | --- | --- | --- | --- | --- | --- | --- |
| Author, year | Was a consecutive or random sample of patients enrolled? | Was a case-control design avoided? | Did the study avoid inappropriate exclusions? | Reference standard | Isthe reference standard likely to correctly classify the target condition? | Were the reference standard results interpreted without knowledge of the results of the index test? | Was there an appropriate interval between index test and reference standard? | Did patients receive the same reference standard? | Were all patients included in the analysis? |
| Rossi,2011 | Y | Y | Y | Pelvic and para-aortic LND by guidelines | N | N | Y | Y | Y |
| Holloway,2012 | Y | Y | N | Complete pelvic and common-iliac LND, aortic LND in high-grade EC | Y | N | Y | Y | Y |
| Manny,2013 | Y | Y | Y | Extended PLND | Y | N | Y | Y | Y |
| Jewell,2014 | Y | Y | Y | Pelvic and para-aortic LND by guidelines | N | N | Y | Y | Y |
| Manny,2014 | Y | Y | Y | Complete pelvic and peri-aortic LND | Y | N | Y | Y | Y |
| Sinno,2014 | Y | Y | N | Complete pelvic and para-aortic LND by guidelines | Y | N | Y | Y | Y |
| Paley,2016 | Y | Y | Y | Pelvic and peri-aortic LND if high risk | N | N | Y | Y | Y |
| Ehrisman,2016 | Y | Y | Y | Complete pelvic LND or Memorial Sloan Kettering algorithm | Y | N | Y | Y | Y |
| Chennamsetty,2016 | Y | Y | N | Extended PLND | Y | N | Y | Y | Y |
| Beavis,2016 | Y | Y | Y | Complete pelvic LND, para-aortic LND at surgeon discretion | Y | N | Y | Y | Y |
| Hagen,2016 | Y | Y | N | Surgeon-discretion LND or Memorial Sloan Kettering algorithm | N | N | Y | Y | Y |
| Eriksson,2017 | Y | Y | Y | Memorial Sloan Kettering algorithm | N | N | Y | Y | Y |
| Mendivil,2017 | Y | Y | Y | Complete pelvic LND, para-aortic LND by guidelines | Y | N | Y | Y | Y |
| Harke,2018 | Y | Y | Y | Extended PLND | Y | N | Y | Y | N |
| Rajanbabu,2018 | Y | Y | N | Pelvic and para-aortic LND by guidelines | N | N | Y | Y | Y |
| Renz,2019 | Y | Y | Y | Complete pelvic LND, para-aortic LND by guidelines | Y | N | Y | Y | Y |
| Rozenholc,2019 | Y | Y | Y | Pelvic and para-aortic LND by guidelines | N | N | Y | Y | N |

LND, lymph node dissection; PLND, pelvic lymph node dissection


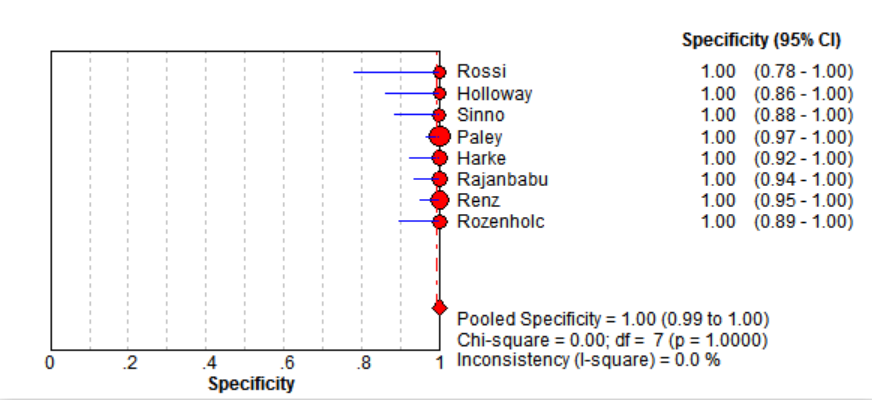


a.


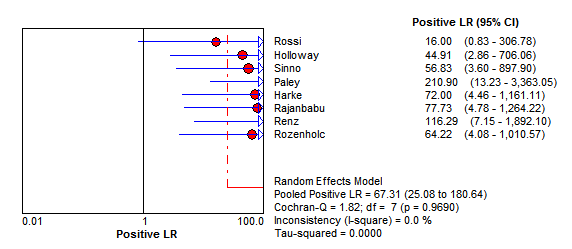


b.


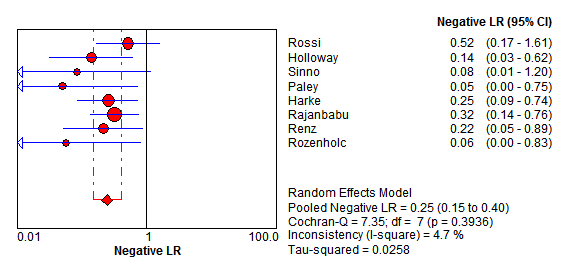


c.


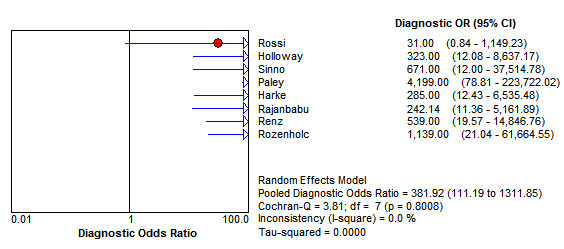


d.

**Appendix Figure 1.** Forest plot of pooled specificity, PLR, NLR, DOR of SLN detection and 95%Cl in SLN mapping

PLR, positive likelihood ratio; NLR, negative likelihood ratio; DOR, diagnostic odds ratio; SLN, sentinel lymph node
